# The Development of a Hypertension Prevention and Financial-Incentive mHealth Program Using a “No-Code” Mobile App Builder: Development and Usability Study

**DOI:** 10.2196/43823

**Published:** 2023-04-05

**Authors:** Amanda Willms, Ryan E Rhodes, Sam Liu

**Affiliations:** 1 School of Exercise Science, Physical and Health Education University of Victoria Victoria, BC Canada

**Keywords:** mobile health, mHealth, usability study, financial incentive, physical activity, mobile phone, smartphone

## Abstract

**Background:**

Regular physical activity (PA) is a key lifestyle component for hypertension prevention. Previous studies have shown that mobile health (mHealth) apps can be an effective tool for improving PA behaviors. However, adherence to and poor engagement with these apps is a challenge. A potential solution to overcome this challenge may be to combine financial incentives with innovative behavior theory, such as the Multiprocess Action Control (M-PAC) framework. Currently, there is a lack of PA financial incentive–driven M-PAC mHealth programs aimed at hypertension prevention.

**Objective:**

We aimed to describe the process of developing an 8-week mHealth PA and financial-incentive hypertension education program (Healthy Hearts) and to evaluate usability of the Healthy Hearts program.

**Methods:**

The first 2 stages of the Integrate, Design, Assess, and Share framework were used to guide the development of the Healthy Hearts program. The development process consisted of 2 phases. In phase 1, the research team met to discuss implementing the M-PAC framework to adopt an existing web-based hypertension prevention program to a mobile app. The app was developed using a no-code app development platform, Pathverse (Pathverse Inc), to help decrease overall development time. In phase 2, we created a prototype and conducted usability testing to evaluate lesson 1 of the Healthy Hearts program to further enhance the user experience. We used semistructured interviews and the mHealth App Usability Questionnaire to evaluate program acceptability and usability.

**Results:**

Intervention development among the research team successfully created an 8-week financial-incentive hypertension education program for adults aged 40-65 years who did not currently meet the Canadian Physical Activity Guidelines (<150 minutes of moderate to vigorous PA per week). This program lasted 8 weeks and comprised 25 lessons guided by the M-PAC framework. The program used various behavior change techniques to further support PA adherence. Usability testing of the first lesson was successful, with 6 participants recruited for 2 rounds of testing. Feedback was gathered to enhance the content, layout, and design of the Healthy Hearts program to prepare the mHealth program for feasibility testing. Results of round 1 of usability testing suggested that the content delivered in the lessons was long. Therefore, the content was divided into multiple lessons before round 2 of usability testing, where feedback was only on design preferences. A minimum viable product was created with these results.

**Conclusions:**

The iterative development process and the usability assessments suggested by the Integrate, Design, Assess, and Share framework enabled participants to provide valuable feedback on the content, design, and layout of the program before advancing to feasibility testing. Furthermore, the use of the “no-code” app development tool enabled our team to rapidly make changes to the app based on user feedback during the iterative design process.

## Introduction

### Background

Hypertension is the leading risk factor for cardiovascular disease and death in the world [1]. The prevalence of hypertension in Canada is comparable with that in the United States and other high-income countries [2], with 25% of Canadian adults currently diagnosed with hypertension and a lifetime incidence of developing hypertension at 90% among adults [3]. Therefore, it is critical to develop effective and scalable hypertension interventions for cardiovascular disease prevention.

Regular physical activity (PA) is a key lifestyle factor to reduce resting blood pressure and prevent hypertension [4]. However, ≥80% of both Canadian and American adults are not meeting the PA guidelines of 150 minutes of moderate to vigorous PA (MVPA) [5,6]. The prevalence of physical inactivity has further increased because of the COVID-19 pandemic [7]. The landscape of traditional in-person PA programs aimed at preventing hypertension has evolved since the COVID-19 pandemic and has created an increased barrier to accessing in-person PA programs [8]. Thus, there is an increasing need to develop effective internet-based health interventions with smartphone apps as a key tool for delivery.

With the recent technological advancements and the adoption of smartphones, mobile health (mHealth) apps have shown the potential to deliver scalable and personalized PA programs [9,10]. However, many mHealth apps suffer from poor engagement [11] and behavioral adherence [12,13]. One strategy to combat these limitations is to ground the mHealth app in behavior change theory. The Multiprocess Action Control (M-PAC) framework addresses what is known as the “intention-behavior gap” through various constructs, including intention formation, action control (adoption), and action control (maintenance) [14-16]. One’s progression through these constructs is further strengthened by behavior change techniques (BCTs). The initial constructs of M-PAC (ie, instrumental attitude and affective judgment) tend to be based on BCTs categorized as social support, natural consequences, and antecedents to behavior. Using techniques that shift an individual’s thinking about completing a behavior, that is, providing information about health consequences and effective action planning, can aid in the shift of one’s thoughts to see a greater benefit than punishment in completing PA [14,17]. Although not addressed by M-PAC, another BCT that can be used to initiate the reflective processes is a material incentive for behavior. This BCT is congruent with M-PAC, as material incentives are a means of increasing the outcome expectation of performance. As one progresses through an intervention grounded in M-PAC, regulatory processes are strengthened with BCTs such as goal setting and self-monitoring.

Financial incentives are a BCT that may assist in adding to the instrumental benefits of PA participation, as health benefits have been criticized as being too distal to an outcome to motivate short-term PA participation [18-20]. Thus, mHealth interventions coupled with financial incentives may be a potential method for overcoming these challenges. Previous systematic reviews have shown that financial incentives can be an effective strategy to improve lifestyle-related behaviors and promote program engagement [21,22]. However, there is a lack of PA-based and financial incentive–based hypertension prevention mHealth interventions tailored for the Canadian population. Such incentive-based mHealth interventions can be critical for a population-based hypertension prevention strategy.

Usability testing is essential for app development to ensure success [23]. To efficiently conduct usability testing and to ensure the app is relevant and useful to the end users, we have used the Integrate, Design, Assess, and Share (IDEAS) framework to guide the development of the app [24]. The IDEAS framework proposes 4 overarching stages to developing effective behavior change interventions: integrate, design, assess, and share [24]. The first stage of the framework, integrate, addresses the following: (1) the needs of the target user, (2) the behavior that the intended user is expected to improve, and (3) grounding this behavior change intervention for the target user in theory. The mHealth hypertension education program used was built on a previous web-based intervention [25]. This provided a foundation for the content in the program, and the expected outcomes relating to improved PA among an adult population have been previously assessed in an iterative process between the research team and the users. In the second stage of the IDEAS framework, the design phase, the research team harnessed the insights from the target user and behavioral theory and (4) envisioned strategies for implementing the information into a deliverable; (5) developed prototypes of potential products; (6) collected user feedback and recommendations; and (7) iteratively build a minimum viable product to use in an intervention based on feedback and overall design. After iteratively following the first 2 stages of the IDEAS framework, a content evaluation is recommended to ensure that the content adheres to evidence-based guidelines [26]. Our team used Pathverse (Pathverse Inc) for this study. Pathverse is a no-code app builder platform that supports mHealth research [27,28]. The Pathverse platform consists of a web portal for researchers to create engaging mobile app interventions with “drag and drop” features instead of coding. The content is then instantly displayed on the Pathverse mobile app.

### Objectives

Thus, the objectives of this study were to (1) describe the process of developing an 8-week self-guided PA financial-incentive hypertension program (Healthy Hearts) using the IDEAS framework and (2) evaluate the usability of lesson 1 of the intervention. We hypothesized that the first lesson of the Healthy Hearts program would be usable and acceptable as it was based on a previous web-based intervention [25]. Following this development and usability study, a feasibility study was conducted to determine the preliminary efficacy of the financial-incentive PA intervention using the Healthy Hearts program [29].

## Methods

### Overview

This study used the first 2 stages of the IDEAS framework [24] to guide the development process of the mHealth intervention. Therefore, this study was divided into 2 phases. In phase 1, we focused on intervention planning and development (December 2020 to February 2021) in line with the integrate stage (steps 1-3) to involve active collaboration among the research team to ensure that the design of the mobile app is relevant and useful to all stakeholders. This consisted of 3 meetings to discuss adapting the web-based hypertension management PA program to a mobile app and restructuring the order of content to align with the M-PAC framework (ie, reflective, regulatory, and reflexive phases). In phase 2, we iterated translating the results from phase 1 into a mobile app, created a beta version of the mobile app, and conducted a series of usability tests with the end users to improve the intervention design of the first lesson further (February to March 2021). This phase was in line with stage 2 of the IDEAS framework, design (steps 4-7). In this phase, end users were invited to an internet-based usability testing session, where they were asked to perform a sequence of tasks.

### Ethics Approval

Ethics approval for this study was obtained from the Human Research Ethics Board at the University of Victoria (protocol number 17-361).

### Informed Consent and Participation

All participants provided written informed consent. Each participant was given a unique identifier to maintain anonymity for data analysis. The participants were informed that they would receive a CAD $20 (US $14.57) Amazon gift card for their time.

### Phase 1: Intervention Planning and Development (December 2020 to February 2021)

#### Steps 1 and 2: Empathize With Target Users and Specify Target Behavior

The first step in intervention planning and development was to address the needs of the target user. As the research team adapted an existing web-based 10-week hypertension management program [30] and prior print materials for the content of the 8-week financial incentive–based hypertension management smartphone app (Healthy Hearts), the research team internally assessed whether the existing programs met these needs. The 10-week hypertension management program was selected for adaptation for its proven success in improving PA levels (ie, daily steps) and blood pressure [30]. Furthermore, the research team evaluated the past success of the M-PAC framework in various web-based interventions that promote PA [31-33] and deemed this framework appropriate to guide an 8-week mHealth app.

The second step of the integrate stage is to specify the target behavior to be used in the intervention. To do this, the research team reviewed the current public health guidelines [3,34] and conducted a literature search of empirical evidence to determine the optimal method for delivering an M-PAC PA intervention through a smartphone [31-33].

#### Step 3: Ground in Behavioral Theory

The final step in the integrate stage of the IDEAS framework is to ground the intervention in behavioral theory. The M-PAC framework was selected as it recognizes 3 stages of the formation of motivation to conduct PA while subsequently addressing the BCTs that assist in closing the gap between intention and behavior. An additional behavior strategy identified by the research team incorporated financial incentives into the M-PAC intervention. Furthermore, the research team explored evidence on past financial-incentive PA interventions and the type of PA to incentivize (ie, daily steps, MVPA, or leisure-time PA) to complement the M-PAC framework as a means of increasing the outcome expectation of performance [35,36]. With these considerations in mind, the research team explored and strategized how the ideas generated in steps 1 and 2 align with the M-PAC framework and BCTs.

### Phase 2: Iterative Design and Usability Testing (February to March 2021)

#### Steps 4 and 5: Ideate Implementation Strategies and Prototype Potential Products

The fourth step of the IDEAS framework, in the design phase, is to ideate implementation strategies. Specifically, we drew on information from various sources, conducted iterative brainstorming sessions to determine the intervention dose, and focused these ideas on app feature implementation and user experience [37]. The research team synthesized the information collected in phase 1 and proposed innovative solutions to modify the original 10-week program. The research team met to discuss content modifications and layout designs to transform the program from being web-based to smartphone-compatible.

The fifth step in the design phase is to prototype potential products. To do so, the research team used the Pathverse program. This program enabled the research team to create an 8-week program that consisted of a combination of *cards* in lessons, which contained educational material, surveys, and quizzes [28]. Furthermore, the Pathverse program had self-monitoring tools, and goal-setting features were used.

#### Step 6: Gather User Feedback

##### Overview

Usability testing of lesson 1 was intended to determine how the target user interacts with a mobile-based program and whether the program meets its intended purpose. We used a mixed methods approach to gain an in-depth analysis of the ease of use and satisfaction and the system information arrangement of the Healthy Hearts program [38]. Overall, 2 rounds of usability testing of lesson 1 were conducted so that no new themes emerged from the testing.

##### Participants

Participants were recruited through paid Facebook posts on the Digital Health Lab at the University of Victoria’s Facebook page. The advertisements ran from January to March 2021. The advertisements were targeted at the intended inclusion criteria of Canadian adults aged 40 to 65 years. Participants were then screened to confirm that they met other inclusion criteria, including access to a smartphone (Apple or Android) with internet, and to confirm that they did not meet the Canadian Physical Activity Guidelines of 150 minutes of MVPA per week [34]. As physical inactivity is a risk factor for developing hypertension [1], this population was deemed appropriate to test the first lesson of the Healthy Hearts app; therefore, current blood pressure measurements were not an inclusion criterion.

##### Procedure

In total, 2 rounds of usability testing were conducted over video chat owing to the COVID-19 pandemic. Individuals were informed that they were allowed to use a nickname or a substitute name and that having their camera on was optional. The individual was informed that this meeting would take approximately 45 minutes and that they must take this meeting from their smartphone, and the individual was asked to download the mobile app, Pathverse, in advance. The meeting was recorded via Zoom local recording along with notetaking by the interviewer throughout the meeting. Audio files were saved to the University of Victoria’s Digital Health Lab secure drive once downloaded following the meeting. Participants were asked to share their phone screen during the video call, so the researchers could follow along with the experience in real time.

Participants were asked to complete a series of goal-oriented tasks to gather user feedback. These tasks included app log-in, completing a lesson, connecting a fitness device to the app’s self-monitoring tools, and setting a personal goal on the app. Participants were asked to “think aloud” as they completed the tasks to further capture their ongoing thought processes while they are using the program as well as any difficulties they may experience [39]. The participants were informed that they were allowed to ask questions but that they may not immediately receive answers to their questions for goal-oriented tasks to follow their full thought processes during troubleshooting, unless they explicitly requested. This was done to help minimize any disruptions to the spontaneous thoughts generated during task execution. Any problems encountered during the tasks were documented. During and after each task, individuals were asked about the esthetics, features, and relevance of the content they were viewing.

The second task was to complete the first lesson of the Healthy Hearts program. This lesson addressed the content that would be covered in the 8-week PA program, including earning a financial incentive, and introduced the benefits of PA for overall heart health. This lesson also incorporated both survey and quiz cards so that the user could see the full potential of a lesson on Pathverse. After completing this lesson, individuals were invited to scroll through all lessons offered in the Healthy Hearts program and were invited to share feedback on lesson photos and names. The next task was to access the fitness page of the program and connect the individual’s phone to display selected fitness data. At the time of testing, only Apple iOS devices were able to display their fitness data. The final task was to go to the “Goal Setting” page and set a goal. After the participant completed the goal-oriented tasks, a semistructured interview was conducted to direct open-ended feedback and further understand the thoughts and emotions of the participants. Textbox 1 provides semistructured interview questions.

**Interview questions and responses.**

Question 1. Can you tell me what you liked best about the app?

- Personalization: all participants who tested the personalized program (n=4) appreciated an introduction of the program creator.

Question 2. Can you tell me what you liked least about the app?

- Display: Pathverse was not yet optimized to as many phones, including those with larger font sizes, so words were often cut out.

Question 3. Can you tell me about how easy it was to navigate or our find your way around the app?

- Lesson progression: many participants (n=4) were not under the assumption to swipe left to progress through the lesson.
- Page access: while accessing 3 pages on the app was well responded to, the symbols representing different pages (ie, fitness page) on the menu bar were not clear.

Question 4. Can you tell me about what you thought about the overall look of the app?

- Design: participants all liked the simple visual of the app, including layout and fonts.

Question 5. Can you tell me what you thought about the information provided on the app?

- Heart health: one participant stated that the content was “very clear” and “didn’t use jargon.”
- Financial incentive: all participants were intrigued by a chance to earn money while simultaneously using the app.

Following the semistructured interview, the participant was asked to complete the mHealth App Usability Questionnaire (MAUQ) [40] to quantitatively capture the participant’s perception of the design and content of the app with questions related to acceptability, usability, satisfaction, and system arrangement.

#### Step 7: Build Minimum Viable Product

The end goal of step 7 was to create a functional smartphone app to be used for subsequent feasibility testing. Modifications recommended from step 6 were repeatedly reviewed to ensure accuracy and adherence. Functionality was repeatedly tested by our internal research team, and any technical issues were reported to the Pathverse development team. Any content issues (ie, spelling and grammar) were reported to 1 researcher (AW) who made final changes to the program. In addition, during this step, the research team ensured that the Pathverse program could adequately support multiple research participants and that engagement data could be accurately downloaded from the Pathverse program.

### Statistical Analysis

The 6-step process of thematic analysis was followed to analyze transcripts and conduct a meaningful evaluation of the qualitative data [41] collected during usability testing and was cross-referenced with notes taken by the researcher (AW). Interview data were coded into categories using a codebook. Thematic analysis was chosen because it allowed researchers to flexibly determine themes as they arose. The categories included colors, features, accessibility, use, and feelings. Each code also indicated whether the comment was positive or negative and whether it was related to the content of the program or user interface. These findings were then presented to the research team to critically question the results. This process ensured rigorous assessment of the data, as we transcribed the data verbatim and arrived at a consensus on the themes presented. The sample size for the usability testing was based on previous studies. Usability issues can be detected within the first 3 to 5 participants [42,43]. Data saturation for the interview was determined when no new themes related to intervention usability were identified from the interview [44]. Quantitative data were analyzed using SPSS (IBM Corp) to describe group demographic characteristics. Descriptive analysis was used to summarize the results of the MAUQ [40].

## Results

### Phase 1: Intervention Planning and Development

#### Steps 1 and 2: Empathize With Target Users and Specify Target Behavior

As determined from the meetings with the research team, the mobile-based program will consist of 8 weeks of content structured around the M-PAC framework. The name of the program was chosen to be “Healthy Hearts.” The goal of the Healthy Hearts program was to prevent hypertension in Canadian adults (aged 40-65 years) who were not currently meeting the PA guidelines (150 minutes of MVPA per week). MVPA minutes were selected as the PA metric, as they are a better gauge of the intensity of the activity completed [45] and are aligned with the Canadian public health guidelines [34].

#### Step 3: Ground in Behavioral Theory

The adapted program consisted of 25 lessons developed based on the M-PAC framework [14,17]. The M-PAC framework addresses the intention-behavior gap through the understanding that ongoing reflective processes (ie, affective attitude and perceived opportunity) and regulation processes (behavioral and cognitive tactics to maintain intention focus) are necessary for the intention to become an action. With this strong foundation of both reflective and regulatory processes, the maintenance of behavior is supported by habit and identity, which can be categorized as reflexive processes [14,17,46,47]. Grounded in the M-PAC framework, the 8-week Healthy Hearts program started with intention formation (lessons 1-10), then moved into action control adoption (lessons 11-19), and concluded with action control maintenance (lessons 20-25). A total of 3 lessons were designed to be delivered each week and encompassed the following topics: PA and heart health, goal setting and planning, PA and mental health, building PA opportunities by restructuring the physical and social environment, and exercise habit and identity formation. The BCTs chosen to be used each week for the intervention content aligned with the operational constructs of the M-PAC framework. The list of BCTs [48] used throughout the lessons is presented in Multimedia Appendix 1.

In this phase, we additionally proposed 2 financial-incentive interventions to accompany the Healthy Hearts app. At the time of writing, the conversion rate was CAD $1 (US $0.73). The first financial-incentive design was a pay-per-minute payout, where participants were rewarded CAD $0.02 for each minute of MVPA tracked through the Fitbit (Fitbit Inc), up to CAD $2.50 per week, and CAD $20 for all 8 weeks. The second financial-incentive design was structured as a self-funded investment incentive where participants commit to invest CAD $400 into their health for the duration of the 8-week program. Participants received a percentage of return on this initial investment based on the number of weeks they successfully met the Canadian Physical Activity Guidelines, as recorded by their Fitbit. The maximum return on investment was 5% or CAD $20, and this was rewarded if the participant met the goal for all 8 weeks of the intervention, similar to the pay-per-minute arm. A 5% return rate of investment was chosen as the maximum to align with the annualized Standard and Poor's 500 stock based on the last 50 years [49]. Consensus around the optimal amount of financial incentive necessary to evoke PA adherence is unclear, particularly when incentivizing MVPA minutes; however, research has shown that as little as CAD $0.10 per day can result in increases in PA [35]. To effectively determine whether the self-funded investment incentive was performing as well as the previously proven successful pay-per-minute arm, the research team hypothesized CAD $20 as an appropriate incentive. Although the efficacy of the proposed financial-incentive designs was not explored in this study, the structure of these interventions was confirmed in phase 2.

### Phase 2: Iterative Design and Usability Testing

#### Steps 4 and 5: Ideate Implementation Strategies and Prototype Potential Products

To address implementation strategies for the mobile-based app, the research team iteratively designed various working prototypes to assess the content and layout of the mobile app. During this iterative process, we considered the features, user interaction, and experiences and combined our unique experiences before usability testing. When the research team assessed what we considered to be an adequate layout (ie, spelling and grammar checks, not too much text per page, and minimal technical difficulties), we determined that the product was ready for user feedback.

**Figure 1:**
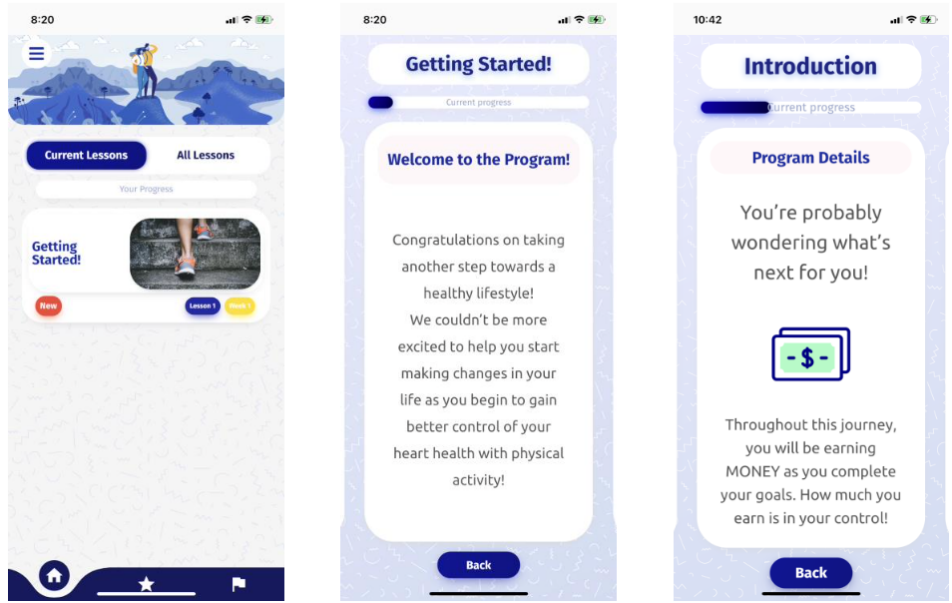
Prototype before usability testing.

The first lesson was created as an overview of the program and titled “Getting Started.” This lesson included instructions on weekly topics, PA guidelines, information about the financial incentive, and how to use their Fitbit. Figure 1 shows a screenshot of the app.

#### Step 6: Gather User Feedback

##### Participant Characteristics

Of the 11 potential participants contacted, 6 (55%) individuals were recruited. The participant sample was 67% (4/6) female, with half of the participants (n=3) being within the age range of 50 to 59 years and all participants being White. All participants had at least 5 years of experience using a smartphone and were experienced in using their smartphone for internet browsing. Table 1 presents participant characteristics.

**Table 1:** Participant characteristics (N=6).

Characteristics		Values, n (%)
**Sex**		
	Male	2 (33)
	Female	4 (67)
	Intersex	0 (0)
**Age group (years)**		
	40-49	2 (33)
	50-59	3 (50)
	60-65	1 (17)
**Ethnicity**		
	White	6 (100)
**Education level**		
	Some college or university	3 (50)
	College or university degree completed	2 (33)
	Graduate degree or higher	1 (17)

In total, 2 rounds of acceptability and usability tests were conducted. Comments made by participants 001 and 002 were from the first round of acceptability and usability testing, and comments from participants 003 through to 006 were from round 2 of testing. The acceptability and usability results from both rounds of testing were cumulated and summarized below.

##### Acceptability of Content and Design

Acceptability questions and feedback determined how the target population used the app and the content they read in their daily life. Findings for the acceptability of the Healthy Hearts program on the Pathverse app were relatively consistent among participants. It was immediately clear after 2 participants completed the first round of usability testing (n=2) that the introductory lesson was too long. The lesson initially consisted of 28 cards (ie, pages of information), and participants 001 and 002 both commented on “losing interest near the end.” This resulted in dividing up the introductory lessons into 2 separate lessons for round 2 of usability testing. From both rounds of testing, 67% (4/6) of the participants “agreed” (score=2) with the following statements: “This app will be useful for my health and well-being,” “I enjoyed reading the content in the lessons,” “I enjoyed using the interactive tools in each lesson,” and “I found the content in each lesson useful.” These statements, along with other acceptability statements that were used in the quantitative analysis, are listed in Table 2.

**Table 2:** Acceptability feedback.

Statement from questionnaire	Values, mean (SD)^a^
The app would be useful for my health and well-being.	2.00 (0.63)
I enjoyed reading the content in the lesson.	2.33 (0.52)
I enjoyed using the interactive tools (ie, survey and quiz features) in the lesson.	2.33 (0.52)
I found the content in the lesson useful.	2.33 (0.52)
I thought the content in the lesson was applicable to me.	2.17 (0.75)
I would imagine that most people who are seeking better health habits would find the content applicable.	2.00 (0.63)

^a^Data from the acceptability questionnaire and item 15 from mHealth App Usability Questionnaire, Usefulness: “1”=strongly agree; “2”=agree; “3”=neutral; “4”=disagree; and “5”=strongly disagree.

There were mixed feelings regarding the self-monitoring fitness page on Pathverse. Overall, 50% (3/6) of participants commented that the star icon to indicate the fitness page “doesn’t mean fitness to me,” as participant 006 stated. On the fitness page, steps were displayed in an accumulation of the past 3 days, the past week, and the past month, a display that participant 002 said, “is simple” and “a good illustration for all abilities.” Participant 004 shared this sentiment, stating “steps is really common and good.” The simplicity of this page was not as positively received, with participant 006 stating “the visual doesn’t mean anything to me.” When probed about what they would like to see displayed on the fitness page, participant 006 stated they “value the distance” and would like to see “steps converted into kilometers.” Participant 004 would “like to see stairs.”

**Figure 2:**
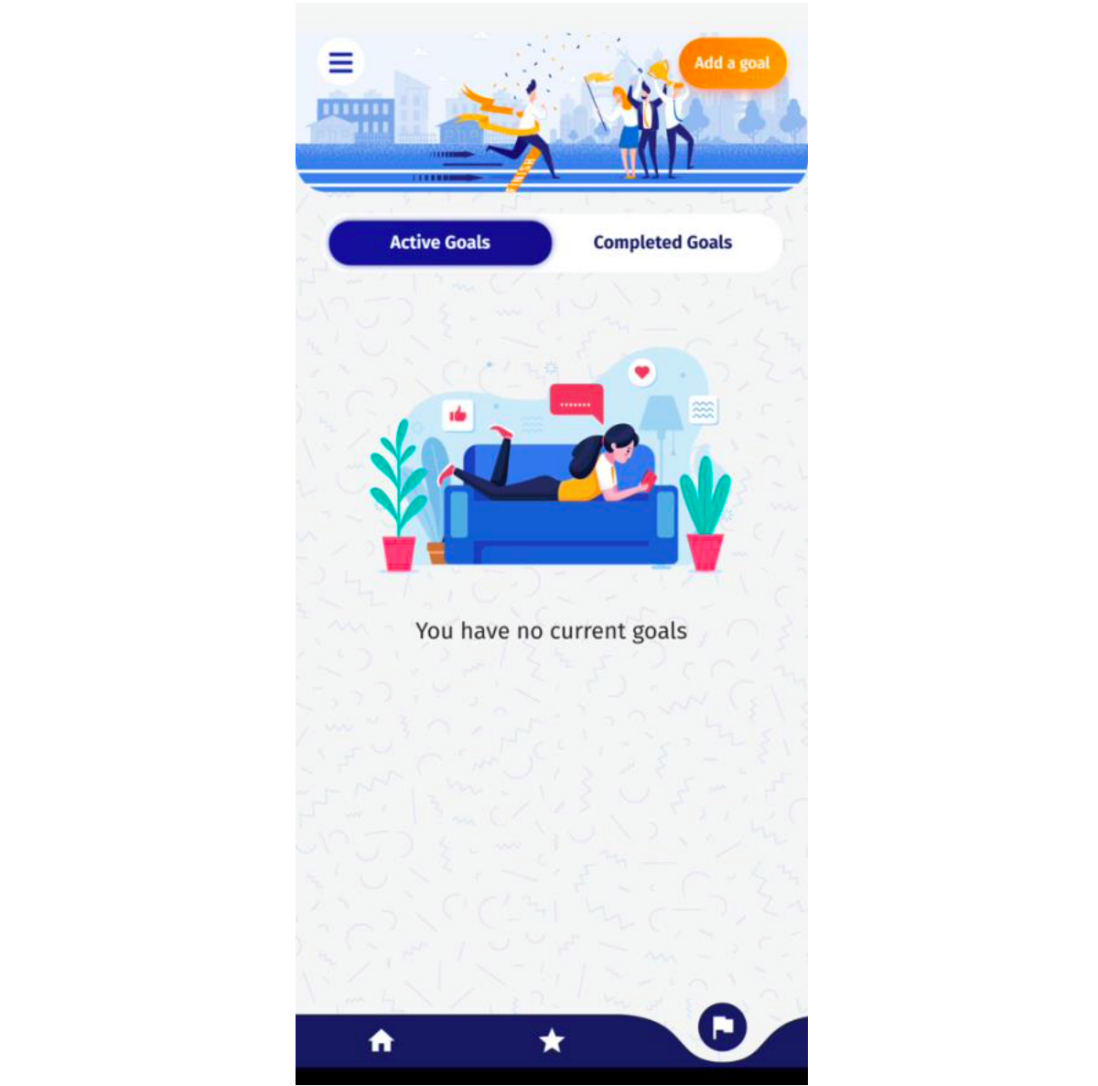
The original Goal Setting page.

Similar to the fitness page, the icon that depicted the Goal Setting page (a flag) “did not mean goals” to participant 006. All participants (6/6, 100%) commented on the location of the “Add a Goal” button on the page. Participant 005 mentioned that the button “is a little bit hidden away” and “kind of got lost” in the header image. Furthermore, “more pathway to setting a goal” was a suggestion from participant 004, and participant 003 suggested addressing “when goal setting will be taught in the program” so the user is aware. Figure 2 shows the original Goal Setting page compared with the updated page.

##### Ease of Use and Satisfaction

Ease of use and satisfaction examined the extent to which the app was an effective, efficient, and satisfactory platform for delivering a heart health education program to those at risk of developing hypertension. All data under the theme “usability” and positive-coded qualitative data were analyzed to supplement the quantitative scores from the MAUQ_Ease of use and satisfaction. Quantitative data analysis supplemented the qualitative data, with most “agreeing” (score=2) or “strongly agreeing” (score=1) with the items in this category. The statement “The amount of time involved in using this app has been fitting for me” was the most agreeable statement, with all participants (n=6) at least “agreeing” with the statement. The same results were obtained for the statement “I feel comfortable using this app on my own” with all participants (n=6) at least “agreeing” with the statement. One participant, participant 003, exclaimed, “Wow, I am impressed with how user friendly this is!” A list of means and SDs for all statements in MAUQ_Ease of use and satisfaction is reported in Table 3.

**Table 3:** Ease of use and satisfaction from the mHealth App Usability Questionnaire.

Statement from questionnaire	Values, mean (SD)^a^
The app was easy to use.	2.17 (0.75)
It was easy for me to learn to use the app.	1.83 (0.75)
I like the interface of the app.	1.50 (0.55)
The information in the app was well-organized, so I could easily find the information I needed.	2.00 (0.89)
I feel comfortable using this app on my own.	1.67 (0.52)
The amount of time involved in using this app has been fitting for me.	1.83 (0.41)
I would use this app again.	1.83 (0.75)
Overall, I am satisfied with this app.	1.67 (0.52)

^a^Data from mHealth App Usability Questionnaire, Ease of Use and Satisfaction: “1”=strongly agree; “2”=agree; “3”=neutral; “4”=disagree; and “5”=strongly disagree.

##### Usability: System Information Arrangement

The system information arrangement referred to the user interface of the mobile app. Questions and comments relating to the navigation, content and feature arrangement, and general interaction with the Pathverse platform were analyzed under this category. The statement “The navigation was consistent when moving between screens” from the MAUQ_System information arrangement was the most agreed upon statement with 83% (5/6) of participants “agreeing” (score=2) with the statement. A suggestion from participant 005 was to “include a card or a tutorial to explain what each piece of the app does.”

One participant “disagreed” (score=4) with the statement “Whenever I made a mistake using the app, I could recover quickly.” This participant brought forward that their fitness data were not displayed, and they were left with “no direction on how to fix it.” The issue has since been resolved. Means and SDs from the MAUQ_System information arrangement statements are presented in Table 4.

**Table 4:** System information arrangement from the mHealth App Usability Questionnaire.

Statement from questionnaire	Values, mean (SD)^a^
Whenever I made a mistake using the app, I could recover quickly and easily.	2.50 (0.84)
This app adequately acknowledged and provided information to let me know the progress of my action.	2.67 (0.52)
The navigation was consistent when moving between screens.	2.17 (0.41)
The interface of the app allowed me to use all the functions (such as entering information, responding to reminders, viewing information) offered by the app.	2.33 (1.21)
This app has all the functions and capabilities I expect it to have.	2.00 (0.63)

^a^Data from mHealth App Usability Questionnaire, System Information Arrangement: “1”=strongly agree; “2”=agree; “3”=neutral; “4”=disagree; and “5”=strongly disagree.

When participants were asked about the layout of the home page and the lesson details, comments were overall positive. All participants (6/6, 100%) were able to switch tabs from “Current Lessons” to “All Lessons” without difficulty. Comments relating to the lessons, including the lesson name, photo, and tags included in the lesson, participant 001 commented, “I like the pictures that have nature in them, it makes me want to go outside.” Participant 003 had the same sentiment and added that nature photos and those exercising “showed fitness” and they “liked that to feel inspired.” Half of the participants (n=3) commented on the colors of tags associated with the lesson. The tags at the time were blue, yellow, and red. The yellow tag with white text was “hard to read” according to participant 001. This statement was matched by 2 other participants, and participant 003 suggested “using orange instead of yellow to compliment the blue and make it easier to read.”

On the basis of these results, along with the following breakdown of ease of use and satisfaction, system information arrangement, and acceptability, the content and the user interface underwent adjustments to improve (1) accessibility and readability of content and colors, (2) accessibility of the Goal Setting page, and (3) guidance for the Goal Setting page within the lessons. Other key changes included layout and esthetic design of the platform, such as adjusting paragraph spacing, adding images, and hyperlinks to access other health-related information (Table 5).

**Table 5:** Themes, quotations, and program modifications.

Theme with sample quotation (participant ID, round number)		Modifications to the program (program features affected)
**Content**		
	“There’s a lot of words and [cards], I definitely lost interest at the end and would have swiped through if you weren’t watching.” [Participant 001, round 1]	Divided first lesson into 2 shorter lessons (lessons 1-2) Ensured length of all lessons were of similar length (lessons 3-25)
**Personalization**		
	“I love the personalization, saying that you’re here with me in the program is very motivating.” [Participant 004, round 2]	Added more reminders throughout that I am cheering participants on
**User interface layout**		
	“I don’t mind the [number] of words, but the layout [font differences, words cut off] wasn’t always appealing.” [Participant 003, round 2]	Consulted with development team to ensure Pathverse was optimized for various smartphones
**Navigation**		
	“Do I have to keep swiping left to progress?” (Swiped left) “Oh that’s how I keep going.” [Participant 006, round 2]	Consulted with development team to change lesson layout delivery Included forward and backward arrows on each page as alternative options to swiping left and right
**Goal setting**		
	“The add a goal button doesn’t stand out to me.” [Participant 002, round 1]	Consulted with development team to move location of “add a goal button” Moved the button from top right to a more central location
**Duration**		
	“I would like to know how much time to budget for each lesson.” [Participant 005, round 2]	Added a tag that recommends the length of time each lesson will take (ie, 6 minutes)
**Mixed media**		
	“Small icons are nice to break up the words.” [Participant 004, round 2]	Included various icons in the middle of each card to break up the amount of text
**Color**		
	“I love the colour yellow [on the tag] but it is very hard to tell what it says with the white text.” [Participant 003, round 2]	Consulted with development team to change the tag colors Changed yellow tag to orange and white text was much more eligible per the Digital Health Lab team

#### Step 7: Build Minimum Viable Product

Through this iterative design process and the collection of user feedback, the research team was able to efficiently develop a minimum viable product to be tested in a feasibility study. The feedback provided by the end user from 2 rounds of usability testing was implemented in the working version of Healthy Hearts. This feedback included optimizing the content and various design changes to the Pathverse platform. Usability testing revealed minimal technical barriers or user interface challenges.

## Discussion

### Principal Findings

This study was designed to highlight the development and usability of the Healthy Hearts mobile-based program. As such, the first objective of this study was to describe the development process of Healthy Hearts, an 8-week PA, and a financial-incentive mobile app grounded in the M-PAC framework. The second objective was to evaluate the usability of the first lesson of Healthy Hearts. The iterative design process used in the first 2 stages of the IDEAS framework involved in the program development promoted the ability to find and make changes quickly when any issues arose. The evaluation of the first lesson of the Healthy Hearts program used a mixed methods approach with quantitative data through an adapted MAUQ, which was described through qualitative semistructured interviews.

By following the IDEAS framework [24] for developing effective digital behavior change interventions, the research team was able to quickly create iterative versions of the Healthy Hearts program for internal and usability testing. Several key factors have contributed to the success of the development of the Healthy Hearts program through Pathverse. First, an iterative design strategy allowed for previous knowledge from the original web-based program [25] to be translated and effectively tested in this study. Second, the Pathverse platform was a simple and efficient method for creating the first prototype and subsequent changes to the platform. This finding is in line with similar studies that followed the IDEAS framework to efficiently develop web-based PA programs [33,50]. The “drag and drop” features used in the Pathverse platform assisted with the efficiency and simplicity to customize the Healthy Hearts program. This framework also allowed feedback between the user and the research team to improve the user interface and experience. Third, the usability assessment enabled the ability to further improve the Healthy Hearts program into an acceptable mobile app program for future pilot studies.

Usability studies that have used only qualitative data typically only present how and why participants make decisions with mobile apps [51-54] and pure quantitative data usability studies are limited to the questionnaires in their findings [55,56]. Thus, our mixed methods approach greater encapsulated the complex phenomenon of usability testing [57]. Furthermore, by using the Pathverse platform, we were able to rapidly and cost-effectively design a functional lesson for usability testing, a major limitation of past usability studies [58,59]. Although the acceptability and usability testing highlighted some usability issues (ie, too much content, layout, and appearance of certain texts and icons), many positive comments were also received about the content and design of the mobile-based program. This finding is similar to past research, which found usability satisfaction to be approximately 73% [60]. Future studies are warranted to determine intervention feasibility, efficacy, engagement, and impact.

To our knowledge, the Healthy Hearts program is the first financial-incentive hypertension program designed for Canadian adults. The Healthy Hearts program was also grounded in the M-PAC theory to address intention formation and action control [14]. The 2 rounds of the usability study enabled our research team to further improve usability and add additional self-monitoring features beyond steps, advanced features with goal setting (eg, delete a goal, date goal set and achieved, and goal priority), and a tutorial on how to use the app. Although our study sample was small (n=6), it has been documented that as few as 5 participants are adequate number of participants for usability testing to reveal major flaws or bugs in the app [61].

The Healthy Hearts program is the first of its kind to be delivered through the no-code app development platform (Pathverse). This new platform was an efficient tool to adopt a web-based program into a mobile app deliverable and enhance the program with features, including surveys and quizzes. The use of Pathverse enables our team to rapidly prototype and change the design of the mobile app without previous knowledge of software coding. We believe that the use of such “no-code” app development can be useful for future health researchers developing mHealth interventions.

### Limitations

Despite the overall positive feedback received from the study, it did not come without its limitations. One of the main limitations of this study was its sample size. However, previous research has shown that samples of 3 to 5 participants for usability testing are adequate [42,43]. Owing to the restrictions imposed by the COVID-19 pandemic, usability testing of this program had to be done through an internet-based video call. Although it was possible to record the participant’s screen and their audio, the participant’s video was shut off when the screen share was enabled, so capturing the individual’s facial expressions or body language while testing the program was not an option. Furthermore, it was harder to control the environment in the participant’s setting, which led to some distractions and interruptions (ie, pets and doorbells). The demographics of this usability testing was not fully encompassing the population of the desired end users. All participants were White and had at least some postsecondary education, which limits the generalizability of the findings. In addition, only 1 introductory lesson was completed during usability testing. Despite this lesson covering an overview of what to expect in the rest of the lessons in the Healthy Hearts program, it did not allow users to see the content in the remainder of the lessons. Finally, we used convenience sampling to recruit participants, which presents further generalizability issues. Future evaluation of the lesson content may be needed before future feasibility evaluations. However, we were able to capture how participants interact with the app through this single-lesson testing.

### Conclusions

This study described the development process and evaluated the usability of an 8-week financial-incentive hypertension education program delivered through a mobile app for Canadians aged 40 to 65 years who did not meet the PA guidelines. We used the IDEAS framework to guide the development of the mHealth intervention. The iterative development process and usability assessments helped our team to identify areas of improvement (ie, text length, layout, text size, and color) before feasibility and efficacy evaluations. Furthermore, the use of the “no-code” app development platform, Pathverse, enabled our team to rapidly prototype and apply these changes to the app based on participants’ feedback. The use of the IDEAS framework and the “no-code” app development tool should be considered for future mHealth intervention development by researchers.

## Appendix

Multimedia Appendix 1 Outline of intervention content and features.

## Abbreviations

BCT: behavior change technique
IDEAS: Integrate, Design, Assess, and Share
MAUQ: mHealth App Usability Questionnaire
mHealth: mobile health
M-PAC: Multiprocess Action Control
MVPA: moderate to vigorous physical activity
PA: physical activity

## Data Availability

The data sets generated and analyzed during this study are available from the corresponding author on reasonable request.
