# Supplementary material for: The Development of a Hypertension Prevention and Financial-Incentive mHealth Program Using a “No-Code” Mobile App Builder: Development and Usability Study
Source: JMIR Form Res. 2023 Apr 5;7:e43823. doi: 10.2196/43823 (PMC10131686; doi:10.2196/43823)
Supplement: Multimedia Appendix 1 [file formative_v7i1e43823_app1.docx]

**Multimedia Appendix 1.** Outline of intervention content and features.

| Week | Topics covered | M-PAC^a^ framework construct | Behavior change technique used |
| --- | --- | --- | --- |
| 1. Getting started in a PA program | - Canadian PA^b^ guidelines - Introducing relationship between PA and blood pressure | Initiating reflective processes (perceived capability and instrumental attitude) | Action planning (1.4)^c^, information about health consequences (5.1),^c^ material incentive for behavior (10.1)^c^ |
| 1. Reaching your PA goals | - SMART^d^ goals - FITT^e^ principle - Enjoyment with PA | Initiating reflective processes (perceived capability and instrumental attitude) and ongoing reflective processes | Goal setting (1.1)^c^, action planning (1.4)^c^, review behavior goals (1.5)^c^, behavioral practice or rehearsal (8.1)^c^ |
| 1. Progressing your PA goals | - Progressing FITT principles - Hypertension and cardiovascular disease prevention with PA | Initiating reflective processes (perceived capability) | Goal setting (1.1)^c^, self-monitoring of behavior (2.3)^c^, information about health consequences (5.1)^c^, graded tasks (8.7)^c^ |
| 1. Your emotions and PA | - Mental benefits of PA - Loneliness and heart health (COVID-19) - Responding to PA-avoiding triggers - Developing trigger management strategies | Ongoing reflective processes (affective attitude) | Goal setting (1.1)^c^, self-monitoring (2.3)^c^, monitoring of emotional consequences (5.4)^c^, behavioral practice or rehearsal (8.1)^c^, distraction (12.4)^c^ |
| 1. Your support system and PA | - 4 pillars of social support - Identifying social support network | Ongoing reflective processes (perceived opportunity) | Goal setting (1.1)^c^, self-monitoring (2.3)^c^, social support (practical; 3.2)^c^, social support (emotional; 3.3)^c^, behavioral practice or rehearsal (8.1)^c^ |
| 1. Your environment and PA | - Creating a positive internal environment to promote PA motivation - Outdoor environment and effect on PA - Changing environment to promote PA | Ongoing reflective processes (perceived opportunity) | Goal setting (1.1)^c^, self-monitoring (2.3)^c^, prompts or cues (7.1)^c^, behavioral practice or rehearsal (8.1)^c^, restructuring the physical environment (12.1)^c^, adding objects to the environment (12.5)^c^ |
| 1. Slippery slope and lifestyle change | - Exercise identity introduction - Balancing responsibilities to maintain PA | Regulation processes and reflexive processes (identity and habit) | Goal setting (1.1)^c^ self-monitoring (2.3)^c^, behavioral practice or rehearsal (8.1)^c^, habit formation (8.3)^c^ |
| 1. Staying motivated after this program | - Getting unstuck during times of relapse - Making informed choices to promote quantum change - Self-monitoring strategies to continue | Regulation processes, reflexive processes (identity) | Goal setting (1.1)^c^, self-monitoring (2.3)^c^, behavioral practice or rehearsal (8.1)^c^, incompatible beliefs (13.3)^c^, valued self-identity (13.4)^c^ |

^a^M-PAC: Multi-Process Action Control.

^b^PA: physical activity.

^c^The numbers in brackets indicate the behavior change technique for reporting in behavior change interventions [48].

^d^SMART: Specific, Measurable, Achievable, Relevant, Timeframe

^e^FITT: Frequency, Intensity, Time, Type
